# Disaggregating innovation for sustainable development in ASEAN: Panel evidence on the moderating role of government effectiveness

**DOI:** 10.1371/journal.pone.0344357

**Published:** 2026-03-05

**Authors:** Pureheart Ogheneogaga Irikefe, Mohammad Falahat, Ahmad Danial Zainudin, Ihtisham Ullah, Nohman Khan, Bernard Ojonugwa Anthony

**Affiliations:** 1 Strategic Research Institute, Asia Pacific University of Technology and Innovation, Kuala Lumpur, Malaysia; 2 School of Accounting and Finance, Asia Pacific University of Technology and Innovation, Kuala Lumpur, Malaysia; 3 Department of Economics and Statistics, Kampala International University, Kampala, Uganda; University of Johannesburg, SOUTH AFRICA

## Abstract

With progress toward the 2030 Agenda faltering, many see innovation as a key to sustainable development. The Association of Southeast Asian Nations (ASEAN) bloc represents a unique opportunity to examine how innovation capabilities drive sustainability in diverse economic and developmental contexts. Using panel data for ASEAN countries from 2011 to 2022, this study breaks down innovation into the seven pillars of the Global Innovation Index (GII) and investigates their impact on the Sustainable Development Goal (SDG) Index; with the objective of identifying which GII pillars most strongly predict SDG, while examining the moderating role of government effectiveness and controlling the impacts of gross national income per capita and foreign direct investment. Fixed effects models were used to analyse the data and supplemented by Driscoll-Kraay standard errors, addressing unobserved heterogeneity and cross-sectional dependence. Results reveal that only Institutions and Infrastructure pillars exert a consistently positive impact on SDG performance. In contrast, Creative Outputs have a negative impact. Importantly, Government Effectiveness reverses the negative impact of Creative Outputs, so that this pillar becomes positive for SDG achievement, without significant moderation of the other six GII pillars when controlling for year effects. In conclusion, these findings contest the efficacy of universal innovation policies and underscore the imperative for nuanced, context‑specific ones. It is recommended that ASEAN governments prioritize institutional and infrastructural investments and develop tailored regulatory frameworks, such as green intellectual property regimes and digital economy standards, to harness the creative economy for inclusive, sustainable growth by explicitly integrating innovation strategies with governance reforms.

## 1. Introduction

The Sustainable Development Goals (SDGs) were adopted in 2015 by all 193 Member States of the United Nations (UN), and represents a worldwide plan to end poverty, lower inequality, and ensure environmental sustainability by 2030 [1,2]. However, recent reports show that progress is faltering; since evaluation after eight years in 2023 reveals only 12% of SDG targets are on track, whereas over 50% are off target, and nearly one-third have regressed [3,4]. Achieving the SDGs demands systemic transformation in resource allocation, technology adoption, institutional reform, and social organization [1,5]. The Asia-Pacific has about 4.7 billion persons, who constitute about 60% of the global population, and adds nearly 37% to global GDP [6,7], hence making the bloc critical in the achievement of global SDGs. Contemporary scientometric reviews assert East Asia, including the Association of Southeast Asian Nations (ASEAN) countries, as contributors of over 21% of the global research on the SDGs, sparkling interest in the region’s economic life and its role in promoting global SDG efforts [6]. Within this bloc, ASEAN, consisting of 10 countries having approximately 678 million people with a combined GDP of US $4 trillion, spans from high income to emerging markets and frontier economies [8]. ASEAN’s heterogeneous development paths thus mean that its collective ability to innovatively accelerate SDG progress, can potentially substantiate its influence into broader regional and global outcomes.

**Fig 1:**
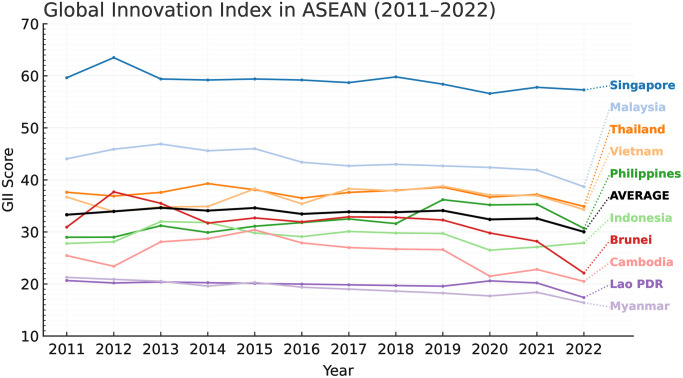
Average GII scores across ASEAN (2011–2022).

**Fig 2:**
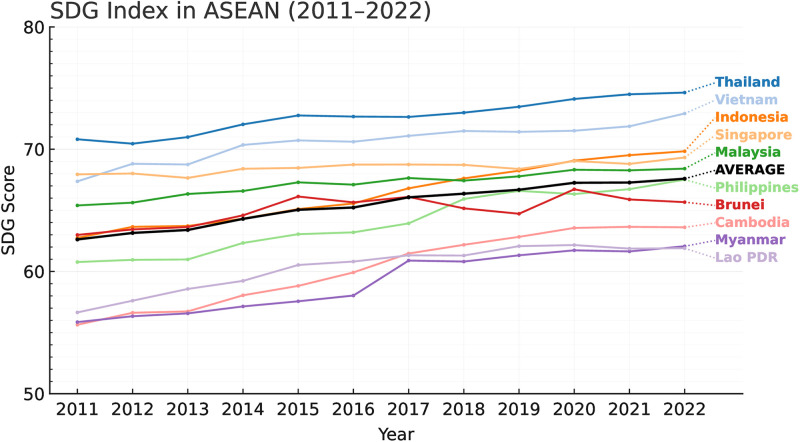
SDG Index scores for ASEAN (2011-2022).

Innovation, which includes new products, processes, business models, and changes in institutions, is enshrined in SDG 9: “Industry, Innovation and Infrastructure,” and is a key element of sustainable development [4,9]. Multidimensional analyses corroborate that innovation simultaneously drives economic development, social justice, and environmental sustainability [10–12]. In ASEAN, their average Global Innovation Index (GII) score devolved from 33.31 in 2011 to 30.02 in 2022, yet the high income and emerging economies like Singapore, Malaysia, and Thailand together account for about 60% of the region’s innovation capacity; leaving middle income and frontier economies like Cambodia (25.8), Myanmar (19.9) and Lao PDR (19.2) well below the ASEAN average as depicted in Fig 1; specifically, in R&D, tertiary education, and ICT infrastructure [6]. Similarly, the regional average SDG Index rose from 62.6 in 2011 to 67.6 in 2022, mainly due to middle-income countries’ progress, as depicted in Fig 2. However, scholars point out that pandemics and disruptions in global trade may soon erode the gain in development [12]. More so, between 2020 and 2021 SDG Index plateaued at 67.3 (see: Fig 2), mainly due to disruptions in healthcare and education access, thus cancelling out earlier gains and subjecting the social infrastructure of the region to serious weaknesses [6].

Importantly, rising averages in innovation or SDG indices do not guarantee equitable development, where structural hurdles persist throughout a region [13,14]. Income inequality in ASEAN remains relatively high; extant studies show that the gaps within the region are larger than those in East Asia’s advanced economies [6,15]. Relatively, a significant digital divide exists; rural areas and small firms often lack access to broadband and skills, which is a limitation preventing them from fully engaging in new value chains [13]. Zhang et al.’s [16] study in ASEAN digital trade, document substantial disparities in digital trade and innovation capacity across member states finding that innovation capabilities can reduce this divide by opening markets. Meanwhile, Lin [15] and He et al. [13] averred rapid urbanization and industrialisation have raised environmental pressures in some economies, even as aggregate SDG scores climb. Together, these trends indicate that without inclusive institutions, growth driven by innovation may further increase existing gaps. More so, recent systematic reviews of SDG progress warn that political and institutional factors, and not just GDP, determine whether innovation translates into broader well-being [2,6,17].

To unpack these nuances in ASEAN whilst drawing lessons relevant to other regions, it is pertinent to disaggregate innovation into its component dimensions and parsimoniously incorporate governance quality. The current study addresses two key questions:

1. Which GII pillars are the most significant predictors of SDG Index performance in ASEAN countries?
2. How does government effectiveness moderate the relationships between the GII pillars and national SDG outcomes?

This study granulates the GII into its seven pillars: “Institutions; Human Capital & Research; Infrastructure; Market Sophistication; Business Sophistication; Knowledge & Technology Outputs; and Creative Outputs” [9] and assess their impact on national SDG performance. Government Effectiveness (from the World Bank’s Governance Indicators) is introduced as a moderating variable. Strong governance has been shown to boost the advantages of innovation and investments in human capital [18]. For example, cross-country analyses demonstrate that transparent, accountable institutions not only enhance economic results but also promote human capital development and technological innovation [17]. The empirical framework uses a panel of ASEAN countries from 2011 to 2022, whilst taking cognizance of confounding factors (such as net foreign direct investments (FDI) inflows and gross national income (GNI) per capita) that might also drive development [10] to highlight and policy gaps.

By isolating which dimensions of innovation capacity matter most, and how it interacts with institutional quality, this research contributes to the innovation-sustainability discourse. Albeit several studies have investigated the broad interrelations between innovation and development [10,11,19,20], none have simultaneously parsed innovation into fine-grained components, contingent on governance effects and tested it; delineating the current study’s novelty. The ASEAN focus is particularly instructive: like other emerging regions (for example, parts of Latin America, Africa or the BRICS), ASEAN countries span a development range and share global challenges such as integrating into world trade, digitalisation and climate risks [15,16,20]. Hence the findings on innovation drivers and governance constraints can potentially resonate beyond Southeast Asia. As Khan et al. [10] and Manzoor et al. [12] averred, policymakers worldwide seek to identify which innovation policy frameworks most effectively advance the 2030 Agenda for Sustainable Development while simultaneously promoting inclusive economic growth. By pinpointing the strongest innovation pillar-SDG relationships and demonstrating how government effectiveness amplifies or dampens these links, this research offers targeted insights: its findings provide a framework for ASEAN and other regions to align innovation strategies with the SDG, ensuring that technological progress translates into prosperity for all.

The remainder of this study is organised as follows: Section 2 reviews literature on innovation metrics and the role of governance in affecting SDG achievement. Section 3 describes our data sources, variable construction and empirical strategy, including method of analyses. Section 4 presents the results of interaction effects and robustness checks and discuss our findings. Section 5 concludes with policy recommendations for leveraging innovation, in conjunction with strong institutions.

## 2. Literature review

### 2.1. Empirical review

Contemporary research has progressively analysed how innovation systems shape sustainable development outcomes, although the results have been mixed, it depends on type of innovation and the locality. Manzoor et al. [12] assesses Sustainability Oriented Innovation Systems (SOIS) for 12 economies (2011–2021) using fixed effect (FE) panel models. They conclude that investments in infrastructure and market sophistication yield the highest GDP growth dividends, with knowledge outputs also facilitating expansion and creative outputs having a less direct influence on effects. Their evidence lends support to Schumpeterian claim that building foundation innovation capacities with targeted policy and technology transfer can drive structural economic change. However, their analysis falls short by concentrating on national aggregates and overlooking how specific innovation pillars advance SDGs within diverse regional contexts like ASEAN. Zhao and Shah [5], in contrast, employ random forest regression based on data from 35 OECD countries (2013–2022) to separate the impact of green energy adoption, GDP growth, and aggregate GII on Human Development Index (HDI) outcomes. Explaining over 87% of HDI variability, they state the application of renewable energy is the best estimator, followed by economic production and innovative potential; confirming the Environmental Kuznets Curve (EKC) hypothesis that harmonious growth, clean energy, and innovation together underpin the strongest human development. Yet their model does not account for the institutional and infrastructural barriers common to emerging markets.

In Europe’s eco‑innovation landscape, Sipos and Ionescu [11] employ structural equation modeling on EU‑27 panel data (2013–2019) to break innovation into five stages: inputs, activities, outputs, resource‑efficiency outcomes, and socio‑economic outcomes. They find that only inputs, activities, and outputs significantly drive an Eco‑Innovation Index, which in turn propels performance on 11 out of 17 SDGs. Yet resource‑efficiency and socio‑economic outcomes often lag or even hamper progress, suggesting that policy instruments must be more precisely calibrated to each eco‑innovation stage. Phuong et al. [21] highlight this level of granularity in the case of Vietnam (1991–2020), using dynamic ARDL approaches, that eco-innovation, eco-investment, and green bonds can all foster environmental protection through reduction of CO₂ emissions while propelling the green growth of the economy forward at the same time. This Vietnamese experience showcases how diverse green-finance tools operate in tandem, while the need for institutional change arises in channeling finances toward environmentally sound projects.

Along with green and eco-innovation, studies on overall technological innovation have presents a nuanced narrative. Chaudhry et al.’s [22] study using the Dynamic Common Correlated Effects estimator in East Asia and Pacific nations (1995–2018), shows that while technology offsets methane, nitrous oxide, and ecological footprints in lower-income countries, it surprisingly worsens CO₂ emissions in higher-income countries. At the same time, institutional quality tends to improve environmental outcomes; however, its effect might vary across pollutant types. Along the same lines, Weber et al. [14] conduct quantile regressions on a global panel of 229 countries between 2013 and 2020, confirming strong negative correlation between innovation (using the Global Innovation Index as proxy) and per capita CO₂ emissions—most evident among top emitters. These global results show that the environmental impact of innovation is both strongly conditioned by economic standing as well as the effectiveness of governance systems.

Several studies for ASEAN and emerging markets explore the dynamics of the innovation-emissions nexus. Zhang et al. [16] use panel models for nine ASEAN countries from 2007 to 2021, finding that innovation, through digital readiness, significantly helps sustainable trade, but they could not offer a thorough evaluation of innovation quality. Lin [15] examines five ASEAN countries from 2000 to 2021 via panel corrected standard errors and mixed-quantile regression, unearthing that digitalization, along with capital accumulation, amplifies ecological footprints, but greentech exerts a nonsignificant, albeit positive, impact; this is most likely limited by exorbitant expenditures coupled with low diffusion intensity. Udo et al. [23] confirm the EKC for MINT countries from 1990 to 2023, noting that although greentech innovation, at the commencement, induces enhanced emissions, globalization, with long-term effects, damps this development, mostly when interaction terms, i.e., greentech innovation multiplied by globalization, are considered. Toha et al. [24] construct a proactive Eco‑Innovation Index for energy firms in Malaysia’s corporate sector (2016–2020) and find, via GLS regression, that process innovation most strongly lifts market performance, significantly moderated by board independence; whereas gender diversity on boards has no discernible impact. Gao and Fan [25] extend these themes to the Belt and Road Initiative countries (2002–2019), confirming both the EKC and Innovation Claudia Curve: technological innovation worsens emissions until crossing a threshold, beyond which institutional quality transforms it into an environmental asset.

Within South Asia and MENA, the literate positions governance as a key moderator. Naz and Aslam [18] test the EKC’s boundaries for South Asia (1996–2019), demonstrating that environmental innovation lowers CO₂, while globalization and financial development increase it; governance magnifies only innovation’s positive impacts. Saleem et al. [26] also find for MENA nations (2002–2020) that innovation by itself increases emissions, governance by itself lowers them, but together they lead to both greener and more robust growth. Hence, suggesting that governance needs to transcend a generic “effectiveness” measure to reflect the multi‑dimensional institutional capacities that make innovation become an agent of sustainability objectives. Firm‑level work, such as Khalil et al. [19], also confirms this duality: on the basis of ESG data from 701 firms from ten Asian economies (2015–2019), they demonstrate that conventional R&D increases profitability at the cost of emissions, while environmental innovation provides both financial and environmental payoffs. Hoa et al. [27] repeat this in Vietnam’s high‑tech export economy, where innovation, FDI, and GDP growth all elevate CO₂, though consumption of renewable energy reduces these impacts. These micro‑level findings reinforce the strategic necessity for firms and policymakers, to encourage green R&D through tax incentives, reporting requirements, and capacity building.

### 2.2. Research gap

In spite of the growing body of studies correlating innovation with sustainable development, three distinctive dimensions of insufficiency can be discerned. First, empirical studies often either describe innovation either as a total summation, represented by the GII score parameterized by Weber et al. [14], or divide it into binary typologies of “green” versus “non-green” [18,26]. As Manzoor et al. [12] and Sipos and Ionescu [11] break innovation apart into disparate inputs, activities, and outcomes, a thorough breakdown of all seven GII pillars in a unified regional frame of reference remains lacking. In turn, policymakers are uncertain which specific building blocks of innovation capacity best trigger breakthroughs in multifaceted SDG indices [20,21]. In addition, whereas governance quality was posited to serve a moderating role on innovation effects [22,25], many studies insufficiently examine the role of overall government effectiveness in shaping the relationships between various innovation pillars cum sustainable development outcomes. Existing studies overwhelmingly evaluate governance moderation based on global datasets [5,14] or focus on univariate national contexts [15,27], thus overlooking whether and to what extent government effectiveness can increase (or decrease) each aspect of innovation’s contributary role towards SDG accomplishment in regions that are highly diverse. Furthermore, empirical inquiries into economic blocs such as ASEAN, EU, MENA, OECD amongst others typically apply broad brush panel methods and seldom account for cross sectional dependence, slope heterogeneity, or endogeneity among variables. Consequently, the dynamic interplay between disaggregated innovation inputs, institutional context, and SDG outcomes remains underexplored and yet to be rigorously tested.

To address these interlinked gaps, this study assembles a fine grained ASEAN panel for 2011–2022, disaggregates the GII into its seven pillars, and parsimoniously employs an aggregated Government Effectiveness index as moderator, while controlling for FDI inflows and GNI per capita. Using a fixed effects approach that includes clustered national scale standard errors, this study accommodates cross-sectional dependence, variability of slopes, and endogeneity. It identifies the specific innovation pillars that are most powerful predictors of national SDG Index scores and formally expresses the broad moderating effects of governmental effectiveness on these relationships. This approach yields robust, usable results for reconciling innovation policy with sustainable development outcomes for Southeast Asia and other emerging economies.

## 3. Materials and methods

### 3.1. Theoretical framework

Sustainable development depends as much on how national systems transform resources into well‐being as on the resources themselves [11,20]. Proponents of the endogenous‐growth theory highlights the increasing returns to knowledge and innovation [28,29], while innovation‐systems research shows that innovation dimensions collectively interact to drive social and environmental gains [9,12,30]. Institutional economics theory proponents adds that governance quality both directly boosts productivity and conditions the payoff to innovation [26,31,32]. The current study, therefore, model SDG performance as the output of an augmented Cobb-Douglas function [33], in which the seven GII pillars and government effectiveness jointly determine total factor productivity, enabling identification of both their main and conditional effects on sustainable development outcomes.

In line with this, national SDG performance in ASEAN is modeled as:


$${\text{SDG}}_{it}=A_{it}\;K_{it}^{\alpha }\;L_{it}^{\beta }$$ (1)

where i indexes each ASEAN country and t the year; $K_{it}$ (GNI per capita) and $L_{it}$ (FDI inflows) proxy for capital and labour and α and β are their output elasticities; together with a technology term $A_{it}$, that captures all other influences. Specifically, $A_{it}$ denotes total factor productivity (TFP) in line with Colther and Doussoulin [34].

TFP is then specified as a product of three elements. First, disaggregated innovation capacities enter through the seven GII pillars ${\text{P}}_{k,it}$ each raised to its own elasticity $\beta_{k}$. Second, government effectiveness (${\text{GOV}}_{it}$) exerts a standalone effect with elasticity γ. Third, institutional quality conditions the returns to a subset of pillars j∈S, so that stronger governance amplifies (or, where weak, dampens) their productivity contribution via exponent $\theta_{j}$. Formally:


$$A_{it}=\left(\prod_{k\;=\;\text{1}}^{\text{7}}{\text{P}}_{k,\;it}^{\beta_{k}}\right)\times {\text{GOV}}_{it}^{\gamma }\times {\prod_{j\in S}\left({\text{P}}_{j,\;it}^{\theta_{j}}\right)}^{{\text{GOV}}_{it}},$$ (2)

where S denotes the set of pillars for which governance interactions are considered relevant based on empirical significance (p < 0.05).

Substituting Equation (2) into Equation (1) and taking natural logarithms yields a log-linear specification expressed in the final model Equation (5). Meanwhile, it is pertinent to first capture the main effects only, expressed in the log-transformed baseline model:


$${\text{ln SDG}}_{it}=\alpha_{i}+\sum_{k\;=\;\text{1}}^{\text{7}}\beta_{k}{\text{ln P}}_{k,it}+{\gamma \text{ ln GOV}}_{it}+\delta_{\text{1}}{\text{ln GNI}}_{it}+\delta_{\text{2}}{\text{ln FDI}}_{it}+\varepsilon_{it},$$ (3)

where $\alpha_{i}$ captures country-specific fixed effects, and $\varepsilon_{it}$ is an idiosyncratic error term clustered by country. Equation (3) identifies which pillars ${\text{P}}_{k,it}$ statistically exert significant influence on SDG performance. To avoid overfitting, only pillars with significant $\beta_{k}$ estimates are retained for subsequent interaction analysis. Let the subset of such pillars be represented thus:


$$S=\;\left\{k:\beta_{k\;}\text{in equation}\left(\text{3}\right)\text{ is significant }\left(\text{p}<\text{0}.\text{05}\right)\right\}.$$ (4)

This ensures moderation is applied only to innovation pillars with demonstrated main effects. The final estimating equation includes only those interaction terms where j∈S:


$${\text{ln SDG}}_{it}=\alpha_{i}+\sum_{k\;=\;\text{1}}^{\text{7}}\beta_{k}{\text{ln P}}_{k,it}+{\gamma \text{ ln GOV}}_{it}+\sum_{j\in S}\theta_{j}\left({\text{ln P}}_{j,it}\times {\text{ln GOV}}_{it}\right)+\delta_{\text{1}}{\text{ln GNI}}_{it}+\delta_{\text{2}}{\text{ln FDI}}_{it}+\varepsilon_{it}.$$ (5)

In this formulation, $\theta_{j}$ captures the marginal conditioning role of governance quality in influencing the elasticity of pillar j. The sequential structure: (1) Cobb-Douglas theoretical baseline grounded in neoclassical growth theory [33,34]; (2) total factor productivity (TFP) decomposition with innovation pillars’ disaggregation—motivated by endogenous growth theory [28,29] and innovation systems theory [30]; (3) main-effects estimation consistent with empirical growth modeling [34] and; (4) selective pillar interaction model informed by institutional economics [31,32], anchors the empirical strategy in a robust theoretical foundation. The moderated fixed-effects model obtained in equation (5) adopts a parsimonious approach, which estimates governance interactions for significant innovation pillars only. This specification eschews overfitting and collinearity issues as well as enabling simpler interpretation of interactive effects. Country fixed effects ($\alpha_{i}$) are included to address both prospective heterogeneity across countries and endogeneity in the SDG-innovation link, and standard errors are country-clustered, yielding inference that is resistant to arbitrary autocorrelation and heteroskedasticity across panels [35,36].

### 3.2. Data description

The analysis uses a balanced panel of 10 ASEAN countries between 2011 and 2022. SDG index (SDG) serve as the dependent variable and key covariates include the seven GII pillars disaggregated (P1-P7), government effectiveness (GOV), gross national income per capita (GNI), and foreign direct investment inflows (FDI). Missing observations (all variables < 5%) were imputed via linear interpolation [37], which preserves trend structure without introducing undue bias when gaps are short and random [38]. All variables are transformed to natural logarithms to mitigate heteroskedasticity, reduce the influence of outliers, and improve the distributional properties of the estimators [34,35]. Table 1 shows the variable names, definitions, and sources.

**Table 1:** Description of Data.

Variable	Definition & Role	Source
SDG Index	Composite index of social, economic, and environmental performance	SDG Index [4]
P1 (Institutions)	Political stability, regulatory quality, rule of law, business-environment indicators	Global Innovation Index [39]
P2 (Human Capital & Research)	Education enrolment, researcher density, R&D expenditure (% GDP)	WIPO and Cornell University [39]
P3 (Infrastructure)	ICT access/use, energy and transport infrastructure quality, ecological sustainability	WIPO and Cornell University [39]
P4 (Market Sophistication)	Ease of credit, venture-capital availability, trade integration	WIPO and Cornell University [39]
P5 (Business Sophistication)	Knowledge-worker density, university-industry collaboration, knowledge absorption	WIPO and Cornell University [39]
P6 (Knowledge & Tech Outputs)	Patent applications, scientific publications, innovation impact metrics	WIPO and Cornell University [39]
P7 (Creative Outputs)	Trademark and design registrations, creative-goods exports, digital creativity indices	WIPO and Cornell University [39]
GOV	Government Effectiveness: quality of civil service, policy formulation and implementation	Worldwide Development Indicators
GNI	Gross National Income per capita (Atlas method, current USD)	Worldwide Development Indicators
FDI	Net foreign direct investment inflows (% GDP)	Worldwide Development Indicators

**Source:** Authors’ compilations.

### 3.3. Empirical strategy and estimation

Identification follows a fixed effects (FE) estimator, consistent with the theoretical model in Equations (1)-(5), where SDG performance is a log-linear Cobb-Douglas function of disaggregated innovation inputs (GII pillars), governance quality, and their interactions. Estimation proceeds in two stages: the baseline model (Eq. 3) captures main effects, while the moderated model (Eq. 5) incorporates interaction terms only those pillars with significant coefficients in Equation (4), maintaining parsimony and minimising overfitting given the moderate panel (N = 10, T = 12, NT = 120). FE is preferred over random effects (RE) because of the heterogeneity persistent in ASEAN countries, such as institutional legacy and governance regimes [8,15,20], which likely correlate with regressors, violating the RE exogeneity assumption [40,41]. Country specific intercepts $\alpha_{i}$ absorb these latent effects, and inference relies on country clustered standard errors to accommodate heteroskedasticity and serial correlation.

The core log-linear specification (Eq. 5) thus integrates both direct and conditional innovation-governance effects on SDG performance. Inference employs country-clustered standard errors, whose covariance estimator is given by:


$$\hat{{\text{Var}}_{\text{cluster}}}\left(\hat{\beta }\right)={\left(X^{\prime }X\right)}^{-\text{1}}\left(\sum_{i=\text{1}}^{N}X_{i}^{\prime }{\hat{\epsilon }}_{i}{\hat{\epsilon }}_{i}^{\prime }X_{i}\right){\left(X^{\prime }X\right)}^{-\text{1}},$$ (6)

where: X is the full NT×K regressor matrix; $X_{i}$ is the T×KT matrix for country i; and ${\hat{\epsilon }}_{i}$ is the T×1 vector of residuals. This estimator accounts for heteroskedasticity, serial correlation, and latent cross-sectional dependence within panels [35,41].

To further mitigate temporal and spatial correlation, Driscoll-Kraay (DK) standard errors are also computed:


$$\hat{{\text{Var}}_{\text{DK}}}\left(\hat{\beta }\right)={\left(X^{\prime }X\right)}^{-\text{1}}\left(\sum_{t=\text{1}}^{T}\sum_{s=\text{1}}^{T}X_{t}^{\prime }{\hat{\varepsilon }}_{t}{\hat{\varepsilon }}_{s}^{\prime }X_{s}\;w_{ts}\right){\left(X^{\prime }X\right)}^{-\text{1}},$$ (7)

where: $X_{t}$ is the N×K regressor matrix at time t, ${\hat{\varepsilon }}_{t}$ is the N×1 vector of residuals, and $w_{ts}=\text{1}-\frac{\left|t-s\right|}{L+\text{1}}$ is the Bartlett kernel [36]. This two-robustness approach offers consistency in the presence of cross-sectional and serial dependence [41].

To verify the fitness of the FE specification against RE, a robust Hausman test (Sargan-Hansen type) is employed. The test ascertains whether FE and RE estimates are consistent under the null hypothesis that there is no correlation between regressors and unobserved heterogeneity [42]. The test statistic follows:


$$H={\left({\hat{\beta }}_{\text{FE}}-{\hat{\beta }}_{\text{RE}}\right)}^{\prime }{\left[\hat{\text{Var}}\left({\hat{\beta }}_{\text{FE}}\right)-\hat{\text{Var}}\left({\hat{\beta }}_{\text{RE}}\right)\right]}^{-\text{1}}\left({\hat{\beta }}_{\text{FE}}-{\hat{\beta }}_{\text{RE}}\right),$$ (8)

where: ${\hat{\beta }}_{\text{FE}}$ and ${\hat{\beta }}_{\text{RE}}$ represents vectors of estimated coefficients from the FE and RE models, respectively; and $\hat{\text{Var}}$ (⋅) denotes their corresponding covariance matrices. This robust version accommodates heteroskedasticity and clustered errors [41,43]. All empirical analyses are implemented in Stata 19 in tandem with Cameron and Miller [41] and Hoechle [35] guidelines.

For visualizsation, in the bid to show and interpret interaction terms, we computed average marginal effects (AMEs) using the model’s estimated variance-covariance matrix and plotted them with 95% CIs. Specifically, after the moderated FE models (with year dummies), we used margins to obtain AMEs of the significant moderated GII pillar across the observed range of GOV, with all regressors mean-centered. Confidence intervals inherit the robust DK specification. Plots were equally generated in Stata 19 with the aid of ‘margins’ and ‘marginsplot’ function.

## 4. Results and discussions

### 4.1. Descriptive statistics and correlations

The descriptive statistics for the current study variables’ raw data are presented in Table 2. The SDG within-country standard deviation of the medium value (1.87) but increased between-country standard deviation (4.55) indicate asymmetrical but incremental sustainability performance improvement. Governance effectiveness (GOV) is between –1.69 and 2.28, indicating differences in institutions between ASEAN. GNI per capita averages USD 12,209 but highly varies both between and within countries (USD 990 to USD 66,910). FDI highly varies between –1.74% and 33.30% of GDP. This heterogeneity in the economy supports the addition of GNI and FDI as controls, representing embedded levels of income and capital openness that influence national innovation capacity and sustainability performance.

**Table 2:** Descriptive Statistics.

Variable	Statistic	Mean	Std. dev.	Min	Max	Observations
P1	overall	56.57867	18.23357	25.4	95.9	N = 120
	between		18.40422	35.05219	94.00833	n = 10
	within		5.005759	37.13701	68.22648	T = 12
P2	overall	29.3973	14.86875	11.1	74.7	N = 120
	between		15.27105	14.14402	64.9	n = 10
	within		3.071083	19.64812	39.19729	T = 12
P3	overall	36.51807	13.48216	13.14361	69.5	N = 120
	between		12.88839	19.05714	62.41667	n = 10
	within		5.568917	14.27068	47.55141	T = 12
P4	overall	47.74905	12.89838	23.5	78.7	N = 120
	between		12.21670	33.35299	74.45	n = 10
	within		5.560244	24.94127	61.54127	T = 12
P5	overall	34.18885	14.93020	8.66654	79.1	N = 120
	between		14.42478	10.10357	66.50833	n = 10
	within		5.837004	19.41256	49.66385	T = 12
P6	overall	25.74561	11.68847	4.2	64.9	N = 120
	between		11.55976	12.42252	50.25833	n = 10
	within		3.917213	17.52309	40.38728	T = 12
P7	overall	26.10725	11.10725	2	45.6	N = 120
	between		10.31796	10.19422	41.1	n = 10
	within		5.172190	5.63491	45.33491	T = 12
GNI	overall	12208.75	17746.91	990	66910	N = 120
	between		18459.05	1246.67	56263.33	n = 10
	within		2390.181	4195.417	22855.42	T = 12
SDG	overall	65.41283	4.723837	55.64	74.63	N = 120
	between		4.551601	59.16250	72.67	n = 10
	within		1.874190	60.79617	68.89783	T = 12
GOV	overall	0.19978	0.983066	−1.68502	2.28420	N = 120
	between		1.01781	−1.30614	2.21426	n = 10
	within		0.161987	−0.17910	0.63423	T = 12
FDI	overall	5.97414	6.715593	−1.73947	33.30458	N = 120
	between		6.749323	1.842678	23.98365	n = 10
	within		1.938206	−0.41348	15.29507	T = 12

**Source:** Authors’ computation.

Among the GII pillars in Table 2, Institutions (P1) records the highest average score (56.58), while Knowledge & Technology Outputs (P7) is the lowest (26.11). The standard deviations for most pillars are larger across countries than within them, particularly for P1 and P5, highlighting structural differences in innovation systems and supporting the use of fixed effects to address unobserved heterogeneity. Further insights on the variable characteristics is revealed in Table 3 showing the pairwise correlation matrix.

**Table 3:** Correlation Matrix.

	P1	P2	P3	P4	P5	P6	P7	GNI	SDG	GOV	FDI
**P1**	1.0000										
**P2**	0.5894*	1.0000									
**P3**	0.6994*	0.7872*	1.0000								
**P4**	0.7666*	0.5351*	0.6128*	1.0000							
**P5**	0.5207*	0.7233*	0.6526*	0.5951*	1.0000						
**P6**	0.1944*	0.5913*	0.4739*	0.4825*	0.6509*	1.0000					
**P7**	0.2318*	0.5769*	0.4552*	0.5584*	0.6418*	0.7619*	1.0000				
**GNI**	0.8840*	0.7118*	0.7431*	0.6729*	0.5906*	0.1942*	0.2789*	1.0000			
**SDG**	0.6058*	0.4052*	0.5947*	0.5582*	0.3090*	0.2494*	0.2651*	0.4988*	1.0000		
**GOV**	0.8268*	0.5597*	0.7077*	0.6923*	0.5900*	0.2250*	0.3311*	0.8507*	0.6534*	1.0000	
**FDI**	0.2573*	0.2149*	0.1130	0.3459*	0.3002*	0.2133*	0.1167	0.2525*	–0.1338	0.1449	1.0000

* p < 0.05 (two‐tailed).

**Source:** Authors’ computation.

Table 3 shows that the GII pillars (P1-P7) are positively and significantly correlated with one another (r = 0.45–0.79, p < 0.05), signifying the interlinked structure of national innovation systems. The same also holds true for high correlations being generated between governance effectiveness (GOV) and institutional pillars like Institutions (P1: 0.83), Infrastructure (P3: 0.71), and GNI (0.85), consistent with the institutional-development nexus in growth theory [32,44]. GNI is itself highly correlated with P1 (0.88), P3 (0.74), and GOV (0.85), indicating that national income is likely to co-evolve with governance and innovation. SDG performance is highly related to all the inputs in innovation (e.g., P1: 0.61; P3: 0.59) and GOV (0.65) and GNI (0.50), supporting the core hypothesis that institutional quality and innovation capability drive sustainability performance. Conversely, FDI has no weak or nonexistent relationships, with no correlation with innovation pillars and no significant negative correlation with SDG scores (–0.13) and hence is a suitable control variable rather than a moderator.

### 4.2. Preliminary tests

To explore any potential multicollinearity among the predictor variables and to guide parsimony in the model, an Ordinary Least Squares (OLS) regression with all the pillars of GII, governance, GNI, and FDI as predictors of SDG performance was conducted. This diagnostic step shown in Table 4, precedes the main FE estimation, as VIFs are most straightforwardly computed within a pooled framework [43,45].

**Table 4:** OLS Regression Results with Multicollinearity Diagnostics.

Dependent variable: ln SDG Index (N = 120)						
**Variable**	**Coefficient**	**(Std. Error)**	**t-value**	**p-value**	**95% Confidence Interval**	**VIF**
**Innovation Pillars**						
ln P1	0.0742**	(0.0365)	2.03	0.045	[0.0018, 0.1466]	7.73
ln P2	0.0306	(0.0220)	1.39	0.168	[−0.0131, 0.0743]	5.52
ln P3	0.0563**	(0.0257)	2.20	0.030	[0.0055, 0.1072]	3.98
ln P4	0.0751**	(0.0336)	2.23	0.028	[0.0084, 0.1418]	4.82
ln P5	−0.0464**	(0.0186)	−2.49	0.014	[−0.0832, −0.0095]	3.32
ln P6	0.0150	(0.0158)	0.95	0.345	[−0.0164, 0.0464]	3.77
ln P7	−0.0159	(0.0153)	−1.04	0.302	[−0.0462, 0.0145]	3.56
**Controls**						
ln GNI	−0.0351***	(0.0106)	−3.31	0.001	[−0.0562, −0.0141]	9.68
ln GOV	0.0862***	(0.0185)	4.66	<0.001	[0.0495, 0.1228]	5.20
ln FDI	−0.0215***	(0.0057)	−3.78	<0.001	[−0.0328, −0.0102]	1.36
**Constant**	1.6182***	(0.0471)	34.36	<0.001	[1.5248, 1.7115]	—

**Model Fit Statistics:**

• R-squared = 0.6365

• Adjusted R-squared = 0.6032

• F(10, 109) = 19.09, Prob > F = 0.000

• Root MSE = 0.01998

**Notes:**

• Robust standard errors in parentheses.

***p < 0.01, **p < 0.05, *p < 0.10.

**Source:** Authors’ computation.

Table 4 provides a diagnostic overview for multicollinearity, using a preliminary OLS regression of SDG outcomes on the full set of predictors. While coefficients for P1 (Institutions), P3 (Infrastructure), and P4 (Regulatory Environment) appear positive, and those for P5 (Business Sophistication), GNI, and FDI negative, these are not interpreted substantively at this stage; since it does not account for unobserved heterogeneity or autocorrelation typical of panel data, which can bias standard errors and coefficient estimates [42,43]. Rather, focus is laid on the variance inflation factors (VIFs), all of which are less than the conventional cut-off of 10, indicating no severe multicollinearity problem [45]. This helps to eliminate these predictors from the following FE model, while including interaction terms is selective in order to maintain parsimony and prevent overfitting [41]. The use of a preliminary OLS step is standard in panel data research for diagnosing collinearity, especially when FE models preclude direct VIF computation [41,46].

Furthermore, to verify whether the panel data exhibited contemporaneous correlations across countries, a set of cross-sectional dependence (CD) tests as shown in Table 5, was applied to the residuals of the FE baseline model.

**Table 5:** Cross-Sectional Dependence Diagnostics.

Test	Test statistic	p-value	Reference metric	Decision/ interpretation
Pesaran CD (standard)	5.007	0.0000	–	**Reject H₀:** cross-sectional independence → Dependence present
Frees Q	0.631	–	**Critical values:** 0.10 = 0.2136, 0.05 = 0.2838, 0.01 = 0.4252	Q exceeds all critical values → Dependence present
Friedman	33.723	0.0001	–	**Reject H₀:** cross-sectional independence → Dependence present

**Source:** Authors’ computation.

Table 5 revealed a strong cross-sectional dependence in the ASEAN panel. The presence of such contemporaneous correlations (Pesaran CD = 5.007, p < 0.001; Frees Q = 0.631 > critical; Friedman = 33.723, p = 0.0001) suggests that shocks affecting one of the ASEAN economy may spill over to others; thus, violating the classical independence assumption [15,35]. Consequently, the study employs DK heteroskedasticity and dependence-robust SEs as robustness to ensure reliable inference. Similar testing procedures are recommended by contemporary econometric literature, including recent treatments in Hoechle [35] and Hounyo et al. [47], which affirm these diagnostics as standard practice for macro-panel data.

### 4.3. Main results

#### 4.3.1. Direct effects.

Table 6 reports the results of the baseline FE regressions estimating the influence of innovation capabilities, governance effectiveness, and economic variables on SDG performance across ASEAN countries. Model 1, without year effects, captures within-country variation over time, while Model 2, which incorporates year dummies, additionally controls for ASEAN-wide shocks such as global crises or coordinated policy changes. Both models control for unobserved heterogeneity between countries and adjusts for within-panel correlation by clustering standard errors at the country level. They extend the earlier multicollinearity and CD diagnostics, providing a more robust specification suitable for causal inference in a panel context [41].

**Table 6:** Fixed-Effects Regression Results (Model 1: without year FE; Model 2: with year FE).

	Model 1:			Model 2:		
Variable	Coefficient (Std. Error)	95% CI	p-value	Coefficient (Std. Error)	95% CI	p-value
**Innovation Pillars**						
ln P1	0.0580*** (0.0158)	[0.0267, 0.0893]	0.0000	0.0312* (0.0169)	[−0.0023, 0.0647]	0.0680
ln P2	−0.0051 (0.0121)	[−0.0291, 0.0189]	0.6720	−0.0046 (0.0117)	[−0.0279, 0.0186]	0.6940
ln P3	0.0571*** (0.0105)	[0.0362, 0.0780]	0.0000	0.0284* (0.0157)	[−0.0027, 0.0595]	0.0730
ln P4	0.0019 (0.0147)	[−0.0272, 0.0310]	0.8980	−0.0059 (0.0145)	[−0.0348, 0.0229]	0.6830
ln P5	0.0117 (0.0104)	[−0.0089, 0.0324]	0.2630	0.0190* (0.0100)	[−0.0009, 0.0390]	0.0610
ln P6	−0.0054 (0.0080)	[−0.0214, 0.0106]	0.5030	0.0002 (0.0077)	[−0.0151, 0.0155]	0.9780
ln P7	−0.0163** (0.0066)	[−0.0294, −0.0032]	0.0150	−0.0109 (0.0066)	[−0.0241, 0.0023]	0.1030
**Controls**						
ln GNI	0.0843*** (0.0112)	[0.0620, 0.1066]	0.0000	0.0712*** (0.0131)	[0.0453, 0.0972]	0.0000
ln GOV	0.0097 (0.0136)	[−0.0173, 0.0366]	0.4780	0.0076 (0.0127)	[−0.0176, 0.0328]	0.5500
ln FDI	−0.0000 (0.0030)	[−0.0060, 0.0059]	0.9870	−0.0007 (0.0028)	[−0.0064, 0.0049]	0.8040
**Year Effects (base = 2011)**						
2012				−0.0045 (0.0084)	[−0.0211, 0.0121]	0.5920
2013	–	–	–	−0.0025 (0.0079)	[−0.0182, 0.0133]	0.7560
2014	–	–	–	0.0055 (0.0087)	[−0.0117, 0.0228]	0.5270
2015	–	–	–	0.0131 (0.0090)	[−0.0047, 0.0309]	0.1460
2016	–	–	–	0.0131 (0.0097)	[−0.0061, 0.0322]	0.1800
2017	–	–	–	0.0243** (0.0106)	[0.0033, 0.0454]	0.0240
2018	–	–	–	0.0224** (0.0106)	[0.0014, 0.0435]	0.0370
2019	–	–	–	0.0201* (0.0114)	[−0.0027, 0.0428]	0.0830
2020	–	–	–	0.0324*** (0.0107)	[0.0113, 0.0536]	0.0030
2021	–	–	–	0.0328*** (0.0110)	[0.0109, 0.0546]	0.0040
2022	–	–	–	0.0244* (0.0126)	[−0.0005, 0.0494]	0.0550
**Constant**	3.0014*** (0.1269)	[2.7497, 3.2532]	0.0000	3.2861*** (0.1451)	[2.9979, 3.5744]	0.0000
**Model Diagnostics**						
Within R²	0.7926	–	–	0.8489	–	–
Between R²	0.2982	–	–	0.2938	–	–
Overall R²	0.3104	–	–	0.3260	–	–
F-statistic	38.2200	–	0.0000	23.8100	–	0.0000
corr(uᵢ, Xb)	−0.8669	–	–	−0.7756	–	–
σᵤ (between)	0.1244	–	–	0.0978	–	–
σₑ (within)	0.0148	–	–	0.0134	–	–
ρ (variance due to uᵢ)	0.9860	–	–	0.9815	–	–
F-test (all uᵢ = 0)	F(9, 100) = 85.77	–	0.0000	F(9, 89) = 81.03	–	0.0000

**Notes:**

• Dependent variable: ln SDG Index

• Robust standard errors clustered by country (in parentheses).

• Variance components: σ

*** p < 0.01, ** p < 0.05, * p < 0.10.

**Source:** Authors’ computation.

Table 6 shows that institutional quality (P1) and infrastructure (P3) significantly lead to improvement in SDG performance across ASEAN, whereas creative outputs (P7) exert a negative impact. Introducing year effects in Model 2 slightly attenuates pillar coefficients, suggesting regional shocks: especially during 2017–2019 and most especially during the COVID period (2020–2021) has a positive impact on sustainability outcomes beyond country-specific factors.

To validate the appropriateness of the fixed-effects estimator over a random-effects specification, a robust Hausman (Sargan-Hansen) test [42] was conducted. The test results, shown in Table 7, strongly reject the null hypothesis (χ²(9) = 96.50, p < 0.001), indicating systematic differences between FE and RE estimates. This ensures that the FE model yields more stable and unbiased estimations in the presence of country-specific effects that are correlated with the regressors [41,43].

**Table 7:** Robust Hausman (Sargan-Hansen) test.

Variable	FE (b)	RE (B)	Difference (b – B)	Std. Error
ln P1	0.0580***	0.0322	0.0258	0.0291
ln P2	−0.0051	0.0389*	−0.0440	0.0276
ln P3	0.0571***	0.1432***	−0.0861	0.0194
ln P4	0.0019	0.0480	−0.0461	0.0270
ln P5	0.0117	0.0176	−0.0059	0.0262
ln P6	−0.0054	0.0005	−0.0059	0.0179
ln P7	−0.0163**	0.0063	−0.0226	0.0140
ln GNI	0.0843***	−0.0127	0.0970	0.0295
ln GOV	0.0097	−0.0381	0.0478	0.0301
ln FDI	−0.0000	−0.0215***	0.0215	0.0067

**Test Summary:**

• Test statistic (χ², 9 d.f.) = 96.50

• p-value = 0.000

• **Decision:** Reject the null hypothesis. The fixed effects model is preferred.

**Notes:**

• This is a robust version of the Hausman test (Sargan-Hansen), computed using the ‘sigmamore’ in Stata 19 option to allow for heteroskedasticity and clustered standard errors.

• Although the variance matrix is not positive definite, this is common in panel applications and does not invalidate the test outcome [41,42].

***p < 0.01, **p < 0.05, *p < 0.10.

**Source:** Authors’ computation.

Table 7 showed that baseline FE estimations provide different insights that confirm as well as enhance existing empirical findings. Consistent with Sipos and Ionescu [11] and Manzoor et al. [12], innovation inputs such as Institutions (P1) and Infrastructure (P3) have a significantly positive effect on sustainability outcomes. This corroborates the theory that foundation capabilities, viz. regulatory quality and physical connectivity, are important to achieving sustainable development, as also seen in studies such as Chaudhry et al. [22] and Gao and Fan [25]. However, unlike Sun et al. [20] and Manzoor et al. [12], no proof of a positive significant contribution of Business Sophistication (P5) or Creative Outputs (P7) is found in this study. In Komorowski and Lewis’ [48] view, the negative baseline effect of Creative Outputs could be attributed to “the environmental cost of creative industries,” which tend to increase carbon emissions and electronic waste when unregulated [13]. The nonsignificant or negative coefficients suggest that not all dimensions of innovation equally converge to SDG performance in ASEAN, possibly due to the variation in structural or absorptive capacity [10,27].

Interestingly, GNI turns out to be a positive and significant predictor; deviating from its negative correlation in the pooled OLS regression. This suggests that with adjustment for country-specific heterogeneity, economic wealth makes a positive contribution to SDG attainment [5]. Governance effectiveness, by contrast, turns insignificant under FE, likely due to limited within-country variance, a limitation highlighted by Saleem et al. [26], and Naz and Aslam [18] when theorising institutional stickiness. FDI is insignificant in both models, echoing the findings of Sun et al. [20] and Khalil et al. [19], that traditional FDI inflows are not likely to be aligned with sustainability objectives. This makes policy interventions triggering green FDI and selective eco-innovation important, particularly in sectors with high environmental spillovers [15,23].

#### 4.3.2. Moderation effects.

Building on the baseline FE model, the study investigates how government effectiveness moderates the relationship between innovation pillars and SDG performance. The interaction-based analysis provides an insight into the facilitative role of institutions in strengthening or diluting the sustainability effect of innovation capabilities; extending the institutional complementarity theories argued in Yıldırım and Gökalp [32] and more recently supported by Saleem et al. [26] and Khan et al. [10].

Table 8 presents the FE moderation models (with- and without-year effects), which includes interaction terms between the baseline significant innovation pillars and governance effectiveness so as to examine conditional effects on SDG performance. Both specifications confirm the suitability of the FE estimator; since the high proportion of variance explained by unit effects (ρ = 0.987 in Model 1; ρ = 0.984 in Model 2) shows existence of strong country-specific heterogeneity. The models exhibit good within-fit (R² = 0.8143 and 0.8576, respectively), and the country-clustered standard errors correct for heteroskedasticity and autocorrelation. The year dummies introduced in Model 2 further capture shared regional shocks: such as global economic slowdowns like the COVID-19 pandemic, as well as coordinated ASEAN policy responses in the bid to enhance robustness over time.

**Table 8:** Fixed-Effects Regression with Interaction Terms (Model 1: without year FE; Model 2: with year FE).

	Model 1:			Model 2:		
Variable	Coefficient (Std. Error)	95% CI	Variable	Coefficient (Std. Error)	95% CI	Variable
**Innovation Pillars**						
ln P1	0.0731*** (0.0189)	[0.0356, 0.1106]	0.0000	0.0452** (0.0200)	[0.0054, 0.0850]	0.0260
ln P2	0.0053 (0.0123)	[−0.0192, 0.0298]	0.6690	0.0035 (0.0123)	[−0.0209, 0.0279]	0.7770
ln P3	0.0436*** (0.0123)	[0.0191, 0.0681]	0.0010	0.0250 (0.0164)	[−0.0076, 0.0577]	0.1320
ln P4	−0.0112 (0.0149)	[−0.0408, 0.0183]	0.4520	−0.0159 (0.0152)	[−0.0462, 0.0143]	0.2980
ln P5	−0.0002 (0.0106)	[−0.0214, 0.0209]	0.9820	0.0103 (0.0108)	[−0.0112, 0.0318]	0.3440
ln P6	0.0000 (0.0080)	[−0.0158, 0.0158]	0.9980	0.0033 (0.0079)	[−0.0124, 0.0189]	0.6800
ln P7	−0.0225*** (0.0068)	[−0.0359, −0.0090]	0.0010	−0.0167** (0.0072)	[−0.0311, −0.0023]	0.0230
**Interaction Terms**						
ln P1 × ln GOV	0.1405* (0.0793)	[−0.0169, 0.2978]	0.0800	0.1112 (0.0781)	[−0.0440, 0.2664]	0.1580
ln P3 × ln GOV	−0.0597 (0.0883)	[−0.2350, 0.1156]	0.5010	−0.0405 (0.0859)	[−0.2112, 0.1303]	0.6390
ln P7 × ln GOV	0.1042*** (0.0347)	[0.0353, 0.1731]	0.0030	0.0699* (0.0362)	[−0.0021, 0.1420]	0.0570
**Controls**						
ln GNI	0.0859*** (0.0112)	[0.0638, 0.1081]	0.0000	0.0768*** (0.0136)	[0.0499, 0.1037]	0.0000
ln GOV	0.0116 (0.0132)	[−0.0145, 0.0378]	0.3780	0.0089 (0.0127)	[−0.0164, 0.0341]	0.4880
ln FDI	−0.0010 (0.0030)	[−0.0069, 0.0048]	0.7280	−0.0015 (0.0029)	[−0.0072, 0.0043]	0.6150
**Year Effects (base = 2011)**						
2012	–	–	–	−0.0064 (0.0084)	[−0.0230, 0.0102]	0.4480
2013	–	–	–	−0.0018 (0.0079)	[−0.0175, 0.0140]	0.8260
2014	–	–	–	0.0037 (0.0091)	[−0.0145, 0.0218]	0.6880
2015	–	–	–	0.0113 (0.0092)	[−0.0070, 0.0296]	0.2220
2016	–	–	–	0.0085 (0.0102)	[−0.0118, 0.0288]	0.4070
2017	–	–	–	0.0207* (0.0110)	[−0.0011, 0.0426]	0.0630
2018	–	–	–	0.0177 (0.0110)	[−0.0042, 0.0396]	0.1120
2019	–	–	–	0.0152 (0.0118)	[−0.0082, 0.0386]	0.2000
2020	–	–	–	0.0275** (0.0109)	[0.0059, 0.0492]	0.0130
2021	–	–	–	0.0258** (0.0115)	[0.0030, 0.0487]	0.0270
2022	–	–	–	0.0169 (0.0130)	[−0.0089, 0.0427]	0.1970
**Constant**	3.0371*** (0.1232)	[2.7926, 3.2816]	0.0000	3.2465*** (0.1451)	[2.9580, 3.5349]	0.0000
**Model Diagnostics**						
Within R²	0.8143	–	–	0.8576	–	–
Between R²	0.2893	–	–	0.2872	–	–
Overall R²	0.3041	–	–	0.3164	–	–
F-statistic	32.7300	–	0.0000	21.5700	–	0.0000
corr(uᵢ, Xb)	−0.8658	–	–	−0.8041	–	–
σᵤ (between)	0.1249	–	–	0.1047	–	–
σₑ (within)	0.0142	–	–	0.0132	–	–
ρ (variance due to uᵢ)	0.9872	–	–	0.9842	–	–
F-test (all uᵢ = 0)	F(9, 97) = 89.20	–	0.0000	F(9, 86) = 75.60	–	0.0000

**Notes:**

• Dependent variable: ln SDG Index

• Robust standard errors clustered by country (in parentheses).

• Variance components: σ

*** p < 0.01, ** p < 0.05, * p < 0.10.

**Source:** Authors’ computation.

Of the main impacts, institutional quality (P1) remains positive and significant (Model 1: β = 0.073, p < 0.01; Model 2: β = 0.045, p < 0.05), meaning that a 1% increase in institutional strength is associated with roughly a 0.07–0.05% rise in SDG performance. Infrastructure (P3) also exerts a positive influence (Model 1: β = 0.044, p < 0.01), meaning a 1% improvement in infrastructure corresponds to about a 0.04% increase in SDG outcomes, albeit impact weakens once year effects are introduced. Creative outputs (P7) shows a negative relationship that is consistent (Model 1: β = –0.023, p < 0.01; Model 2: β = –0.017, p < 0.05), which suggests that higher creative intensity exacebate trade off with sustainability gains; in line with baseline findings. Among interaction terms, only P7 × GOV is statistically significant and positive (Model 1: β = 0.104, p = 0.003; Model 2: β = 0.070, p = 0.057), implying that a 1% improvement in governance strengthens the positive contribution of creative outputs to SDG performance by about 0.07–0.10%. In contrast, P1 × GOV and P3 × GOV show the expected signs but lack statistical significance when year effect are introduced. In retrospect, the results suggest that governance moderating role is selective: amplifying specific innovation dimensions rather than exerting a uniform effect across all pillars.

The varying impacts across innovation pillars are consistent with recent findings that the sustainability impact of innovation varies with different contexts [16,19]. The positive P1 (Institutions) coefficient illustrates the way in which the quality of governance (e.g., regulatory institutions, rule of law, political stability), channels innovation to green outcomes [25,26]. Similarly, P3 (Infrastructure) improves SDG performance, which is in line with extant literature that aver energy and digital infrastructures drives sustainable development [12,20]. Conversely, P7 (Creative Outputs) has a negative impact on SDG, suggesting dangers in unregulated creative businesses [13,27]. Most critically, however, the P7 × GOV interaction is positive, implying that good governance can undo these tradeoffs [18,23]. The non-significant interactions for P1 × GOV and P3 × GOV suggest possible maturity thresholds, as noted in Lin [15] and Toha et al. [24], where further governance gains yield diminishing returns in well-established domains.

These differentiated effects imply that ASEAN policymakers must move beyond one size fits all reforms and instead design tailored innovation–governance frameworks. For Institutions (P1), this means constant legal and regulatory reforms to ensure SDG alignment, as noted by Saleem et al. [26] and Gao and Fan [25], increasingly toward sector-specific interventions as soon as baseline governance is established. For Infrastructure (P3), governments should invest in green and energy digital systems, embedding sustainability metrics and technology transfer, as recommended by Manzoor et al. [12] and Sun et al. [20], but recognize that further governance development in this field can have declining returns. For Creative Outputs (P7), the development of specialised regulatory frameworks for digital and creative economies; strengthened intellectual property rights and green content rules, can harness creativity for SDG progress [18]. Finally, the relatively small individual effects of FDI and governance suggest that foreign investment and the quality of institutions must be complemented by innovation policy [14,21], which highlights the importance of whole-of-policy coordination among trade, environmental, and innovation portfolios.

### 4.4. Robustness checks

To test the robustness of our baseline and moderation model estimates, we re-estimated both specifications according to Driscoll-Kraay (DK) standard errors taking cognizance of year effects, which are heteroskedasticity-, serial correlation-, and cross-sectional dependence-robust; common features of macro-panel data structures like ASEAN [35,36]. This approach addresses the potential biases of conventional fixed-effects estimators that are founded upon weaker independence assumptions across panels, thereby enhancing confidence in the stability of the coefficient estimates under more general conditions.

**Fig 3:**
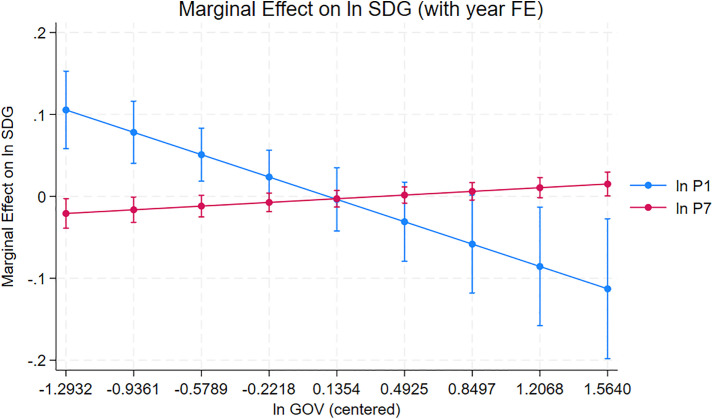
Marginal effects of governance on innovation pillars (with year fixed effects).

Table 9 largely confirms the patterns observed under country-clustered standard errors. Across all specifications, Institutions (P1) and Infrastructure (P3) remain positive and significant predictors of SDG performance, underscoring their foundational roles in the innovation–sustainability nexus. The negative baseline effect of Creative Outputs (P7) persists in the models without year controls in line with Komorowski and Lewis [48], yet under the moderated specifications: especially when year fixed effects are included, the negative pattern weakens. This shift arises because the year effects capture region-wide shocks, such as the pandemic-induced disruptions in 2020–2021 and subsequent recovery in 2022, which heavily affected creative and digital sectors [13,48]. When these shocks are accounted for, and governance interactions are introduced, the P7 × GOV term turns strongly positive, in line with Naz and Aslam’s [18] study that effective governance frameworks can transform creative industries from potential environmental liabilities to become contributors to sustainable development. Thus, government effectiveness appears to cushion the volatility of creative outputs over turbulent periods [26], allowing the sector’s positive spillovers: digital inclusion, cultural exports, and green creative content, to manifest more clearly in the presence of stable institutions. This dynamic moderating pattern will be further illustrated in Fig 3, which presents the marginal-effects plot for Creative Outputs (P7) across varying levels of government effectiveness.

**Table 9:** Driscoll-Kraay FE Estimates for Baseline and Moderated Models.

Variable	Baseline 1 (without year FE)β (SE)	Baseline 2 (with year FE)β (SE)	Moderation 1 (without year FE)β (SE)	Moderation 2 (with year FE)β (SE)
**Innovation Pillars**				
ln P1	0.0580*** (0.0068)	0.0312*** (0.0058)	0.0731*** (0.0122)	0.0452*** (0.0084)
ln P2	–0.0051 (0.0115)	–0.0046 (0.0111)	0.0053 (0.0130)	0.0035 (0.0150)
ln P3	0.0571*** (0.0122)	0.0284** (0.0133)	0.0436*** (0.0089)	0.0250** (0.0115)
ln P4	0.0019 (0.0206)	–0.0059 (0.0233)	–0.0112 (0.0212)	–0.0159 (0.0264)
ln P5	0.0117 (0.0096)	0.0190* (0.0104)	–0.0002 (0.0079)	0.0103 (0.0110)
ln P6	–0.0054 (0.0054)	0.0002 (0.0069)	0.0000 (0.0060)	0.0033 (0.0080)
ln P7	–0.0163 (0.0111)	–0.0109 (0.0099)	–0.0225*** (0.0064)	–0.0167** (0.0061)
**Interaction Terms**				
ln P1 × ln GOV	–	–	0.1405*** (0.0390)	0.1112*** (0.0168)
ln P3 × ln GOV	–	–	–0.0597 (0.0598)	–0.0405 (0.0516)
ln P7 × ln GOV	–	–	0.1042*** (0.0234)	0.0699** (0.0334)
**Controls**				
ln GNI	0.0843*** (0.0071)	0.0712*** (0.0113)	0.0859*** (0.0057)	0.0768*** (0.0075)
ln GOV	0.0097 (0.0100)	0.0076 (0.0093)	0.0116 (0.0096)	0.0089 (0.0100)
ln FDI	–0.0000 (0.0031)	–0.0007 (0.0037)	–0.0010 (0.0021)	–0.0015 (0.0033)
**Year Effects (base = 2011)**				
2012	–	–0.0045 (0.0028)	–	–0.0064 (0.0041)
2013	–	–0.0025 (0.0028)	–	–0.0018 (0.0031)
2014	–	0.0055** (0.0025)	–	0.0037 (0.0031)
2015	–	0.0131*** (0.0025)	–	0.0113** (0.0032)
2016	–	0.0131** (0.0039)	–	0.0085 (0.0050)
2017	–	0.0243*** (0.0044)	–	0.0207** (0.0052)
2018	–	0.0224*** (0.0036)	–	0.0177*** (0.0039)
2019	–	0.0201*** (0.0039)	–	0.0152*** (0.0038)
2020	–	0.0324*** (0.0046)	–	0.0275*** (0.0039)
2021	–	0.0328*** (0.0051)	–	0.0258*** (0.0048)
2022	–	0.0244*** (0.0078)	–	0.0169** (0.0072)
**Constant**	3.0014*** (0.0894)	3.2861*** (0.0915)	3.0371*** (0.0738)	3.2465*** (0.0926)
**Model Fit Statistics**				
F-statistic	F(10,11)=9176.88 ***	F(21,11)=55.10 ***	F(13,11)=17691.28***	F(24,11)=1951.06 ***
Within R²	0.7926	0.8489	0.8143	0.8576

**Notes:**

• Dependent variable: ln SDG Index.

• Estimates derive from FE regressions with DE SEs (maximum lag = 2).

• Each model uses 120 country-year observations across 10 ASEAN countries.

• The inclusion of year dummies (Model 2 specifications) captures region-wide shocks.

Significance levels: *p < 0.05, **p < 0.01, ***p < 0.001.

Source: Authors’ computation.

Fig 3 shows the conditional marginal effects of Institutions (P1) and Creative Outputs (P7) on Sustainable Development across the levels of Government Effectiveness (GOV), as revealed by the moderated fixed effects model including year dummies in the ASEAN bloc. A downward-sloping line shows that as countries move towards better governance, the additional importance of institutional innovation becomes gradually less; this captures maturity effects observed in countries that already practice high governance standards. By contrast, as governance is satisfactory, the negative influence of the creative sector is overturned, making the creative economy a positive factor in SDG performance. These error bars represent a confidence interval of 95% DK robust SEs.

These robustness checks echo the validation approach employed by studies such as Manzoor et al. [12] and Sun et al. [20], where consistent significance across alternative estimators was used to demonstrate the reliability of infrastructure and institutional variables in explaining development outcomes. The findings also validate previous research by Naz and Aslam [18] and Khan et al. [10], whose research proved that innovation outcomes, particularly in creative or digital sectors, rely crucially on institutional congruence.

Together, the findings of the DK specification in the current study affirm both the validity and soundness of the main findings. They affirm that the FE models captures meaningful relationships between disaggregated innovation inputs and SDG outcomes, and that the role of governance in moderating these effects, especially for creative outputs, is not spurious, but rather withstands stricter error assumptions. This empirical reliability strengthens the basis for policy recommendations tailored to sector-specific innovation-governance synergies across ASEAN.

### 4.5. Practical applications

These results point to a practical ordering for ASEAN. Across the FE and DK specifications, the two pillars that most consistently align with higher SDG performance are the foundational ones: Institutions (P1) and Infrastructure (P3). Creative Outputs (P7) is negative in the direct relationship, but the interaction terms show that this downside shrinks as government effectiveness rises, and can turn into a net positive at higher governance levels in line with Table 9 and Fig 3. In other words, the message for practice is not “do more innovation” in general. It is: fix the enabling conditions first, and then scale higher-visibility innovation; especially creative activity, through governance arrangements that keep the social and environmental spillovers under control.

1. Use institutional reform to raise implementation capacity: The institutions-and-development literature has long stressed that predictable rules, credible enforcement, and public-sector capability shape long-run development outcomes [32,44]. In our setting, the positive role of P1 suggests that reforms such as more transparent procurement, faster and rules-based licensing, stronger contract enforcement, and stable policy signals are likely to pay off because they increase the chances that sustainability programmes actually get implemented as designed. Since “government effectiveness” is itself a broad indicator of state capability [31] and is repeatedly linked to development outcomes [17], these reforms should be framed as delivery upgrades rather than administrative tidy-ups.
2. Prioritise infrastructure that removes binding constraints: The robust positive association for P3 implies that infrastructure is not a background condition; it is part of the SDG pathway. For ASEAN, that typically means (i) reliable and cleaner energy systems, (ii) resilient transport and logistics, and (iii) digital connectivity that expands access to services and markets. ASEAN regional evidence shows that energy structure, diversification, and resource efficiency are closely tied to sustainable development trajectories [5,7,20]. Where the digital economy is used as a development lever, the emphasis should be on broad, dependable access rather than narrow, high-end upgrades; as He et al. [13] posited, digitalisation only improves sustainable outcomes when it is paired with real capability and access.
3. It is pertinent that the creative economy be treated as a conditional lever, and should be governed accordingly: Our results indicate that Creative Outputs (P7) can coincide with weaker SDG performance on average, but that the relationship improves as government effectiveness rises. A straightforward reading is that creative-sector growth can produce externalities (energy demand, e-waste, uneven urban impacts, precarious work) unless public institutions are able to set and enforce standards. This concern is consistent with work on the creative and cultural industries that emphasises the need for sustainability-aligned rules and recovery pathways [48]. It also aligns with evidence that governance can change the direction and strength of “innovation → sustainability” relationships in other country settings [18,26]. Practically, this means pairing creative-sector support with enforceable safeguards (for example, energy-efficiency requirements for production facilities and digital services, circularity requirements for devices and equipment, and procurement criteria that reward low-impact creative solutions) and enforcing them through routine compliance rather than one-off campaigns.
4. Be selective about FDI rather than chasing volume: Because FDI inflows do not show a stable relationship with SDG performance in our models, policies that compete mainly on the size of incentives are unlikely to deliver predictable sustainability gains. A more defensible approach is to target capability-building and sustainability-aligned investment: technology transfer, supplier development, and cleaner production requirements, which is where the strongest channels are usually located in related empirical work [25,27].

A simple way to operationalise the four practical applications of the current study is for ASEAN economies to run an “innovation-to-SDG” review each year: track SDG outcomes alongside the specific GII pillars one can move (especially P1 and P3), and check whether improvements in those levers precede changes in SDG performance. Using established SDG and innovation measurement frameworks makes this feasible without inventing new indicators [1,4,39].

## 5. Conclusion and recommendations

This study sets out to disentangle how specific innovation dimensions drive sustainable development across ASEAN’s ten countries from 2011 to 2022, and whether government effectiveness conditions these relationships. By employing FE models with both clustered and DK standard errors, the direct effects of each GII pillar on the SDG performance index were isolated, controlling for income levels and FDI. The findings confirm that Institutions and Infrastructure alone make uniformly strong and positive contributions to SDG impacts, underlining the underpinning role of well-regulated environments, stability of political environments, and well-developed physical and cyber infrastructures. More advanced innovation indicators (Business Sophistication and Knowledge & Technology Outputs) did not significantly impact, whereas Creative Outputs had a uniformly negative direct effect. It is crucial that when compounded by high government effectiveness, the negative baseline impact of the creative economy is turned into a positive force towards sustainability, creating a selective rather than blanket institutional capacity moderation effect.

These relative effects underscore the need for ASEAN region-nonspecific approaches to innovation-governance. Accordingly, three recommendations consistent with the findings of the current study are recommended thus:

1. Governments have to go on strengthening core institutional pillars: This involves upholding rule of law, enhancing regulatory coherence, and ensuring open decision making, and aligning public and private investment with green, resilient infrastructure, led by renewable power and digital connectivity.
2. The creative economy requires bespoke regulatory mechanisms: This encompasses intellectual property systems that reward long-term innovation, “green” codes of content that align cultural production with ecologically rational goals, and digital literacy initiatives that ensure open access to creative platforms.
3. Foreign investment and governance reforms must be explicitly woven into national innovation strategies: Rather than attracting capital indiscriminately, governments should incentivise “green FDI” that brings sustainable technologies, and shape institutional reforms to enhance the economy’s capacity to absorb and deploy innovative solutions for the SDGs.

Taken together, these recommendations provide a practical pathway for translating the study’s evidence into SDG-oriented innovation policy in ASEAN. Nonetheless, despite the robust panel design, this study has limitations. Relying on an aggregate government effectiveness index may conceal the distinct influences of anti corruption, policy implementation, and administrative quality. Although FE control for unobserved, time invariant heterogeneity, endogeneity between innovation inputs and sustainability outcomes remains a potential concern. Future research should employ more disaggregated governance measures and dynamic panel methods or quasi experimental approaches so as to better identify causal pathways. Deeper disaggregation of innovation categories and country specific case studies will more closely calibrate nuanced policy direction to ensure that the innovation potential is translated into sustainable and inclusive advance towards the 2030 Agenda.
